# A Case of Herpes Simplex Virus-1 Encephalitis from a Medicolegal Point of View

**DOI:** 10.1155/2018/3764930

**Published:** 2018-06-10

**Authors:** Alessandro Feola, Anna Mancuso, Mauro Arcangeli

**Affiliations:** ^1^Department of Biomedicine and Prevention, University of Rome “Tor Vergata”, Rome, Italy; ^2^Department of Life, Health and Environmental Sciences, University of L'Aquila, L'Aquila, Italy

## Abstract

We present a case of herpes simplex virus-1 encephalitis (HSVE) and discuss the difficulty of early diagnosis and the possibility of a wrong or delayed diagnosis and treatment of this encephalitis. We show the importance of considering HSVE to pursue every case of suspicious medical liability.

## 1. Introduction

Herpes simplex virus-1 (HSV-1) encephalitis (HSVE) is a rare viral infection of the human central nervous system (CNS) entailing neurological dysfunction. However, it is the commonest infectious cause of sporadic encephalitis. The annual incidence of HSVE worldwide is estimated to be 1–4 cases/1,000,000 [1, 2].

HSVE has a bimodal age distribution, with peak incidences in children less than 3 years old and those aged 50 years or more. Most cases occur in subjects older than 50 years of both sexes. HSVE is difficult to diagnose and has a poor prognosis. Morbidity and mortality are greater if treatment is delayed or inadequate. In these cases, there could be medicolegal consequences, particularly legal liability for medical malpractice and nervous system injury assessment. We report the case of a 60-year-old man with HSVE.

## 2. Case Presentation

A 60-year-old diabetic patient with chronic kidney disease, and treatment with corticosteroids for nephrotic syndrome, came into our emergency room. He presented with fever, dyspnea, and disorientation and was in a fugue state with naming difficulties and aphasia. A complete blood count showed a white blood cell count of 14,630/ml and his C-reactive protein was 1.2 mg/dl (normal range, 0–0.5 mg/dl). A computed tomographic scan was negative for brain injury. Cerebrospinal fluid (CSF) tests were positive: the fluid was turbid, glycorrhachia was 141 mg/dl (normal range, 50–80 mg/dl), proteinorrachia was normal, and the CSF white cell count was 135/UI (normal range, 0–5/UI) with a left shift (90% neutrophils and 10% lymphocytes). The patient was given broad-spectrum antibiotic therapy while awaiting the CSF culture results. Two days later, the patient was comatose, with right hemiplegia. He did not obey simple commands and opened his eyes only after painful stimulation. The CSF culture was negative for bacteria, but viral DNA corresponding to HSV-1 was detected in the fluid. Magnetic resonance imaging detected diffuse signal changes in the cortical and subcortical matter, especially in the frontal-temporal region and the parietal region in both cerebral hemispheres, but particularly in the left hemisphere. Doctors diagnosed herpetic encephalitis. They prescribed acyclovir treatment at a dose of 750 mg/250 ml in 3 h three times a day for 21 days because the patient's glomerular filtration rate ranged from 10 ml/min to 50 ml/min. The patient was moved to a rehabilitation facility after 21 days. At discharge from the rehabilitation facility, the patient had a percutaneous endoscopic gastrostomy (PEG) and experienced hyperactivity of the muscle stretch reflex with extension of the knee on the right side. He was able to move his right foot, extend his right knee, and walk unassisted, but he had some problems with his dynamic balance.

Six months later, the patient underwent brain MRI, which showed a large unenhanced signal alteration. This alteration was consistent with a malacic area of gliosis in the left temporal lobe and to a lesser extent in the left frontal lobe, in the corona radiata, and in the centrum semiovale. There was also ex vacuo dilatation of the right-left ventricle. On the right side, the signal was iperintense in the temporal, frontal, and parietal periventricular white matter.

Ten months after the onset of encephalitis, the patient was treated with PEG for recurrent dysphagia. He had a mask-like face and double incontinence. He was able to obey simple commands but was unable to speak. He had joint limitations in the shoulders, elbows, and hands, with extrapyramidal side effects and pyramidal hypertonia of the upper limbs. The patient had lost all motor function and was totally dependent.

## 3. Discussion

There are two main types of HSVE, primary and recurrent. In more than 70% of cases, HSVE is caused by the reactivation of a latent virus in individuals who have previously been infected. Once reactivation has occurred, viral particles are transported via the anterograde axonal transport via the olfactory and trigeminal pathways to the CNS [3]. In 30% of cases, HSVE is caused by a primary infection, and the virus also reaches the CNS by the olfactory and trigeminal nerves. In immunocompetent hosts, HSVE affects brain regions such as the limbic system, mesial temporal and frontal regions (amygdala, hippocampus, parahippocampal gyrus, temporal uncus, insula, and cingulate gyrus) earlier and most severely. The putamen and basal ganglia are usually unaffected [3].

In many cases, the presentation of encephalitis is monolateral in the beginning, but the lesions gradually become widespread and bilateral, and they affect the contralateral temporal lobe asymmetrically. HSVE sometimes affects the occipital cortex. The lesions are uneven and asymmetrical. Herpetic encephalitis is marked by necrotic-hemorrhagic lesions, with perivascular lymphocytic infiltration in the necrotic tissues and the meninges [4]. These result in broad malacic areas as a result of tissue destruction and secondary atrophy [5]. Infected patients develop prodromal symptoms, such as malaise, fever, headache, and nausea, followed by the acute or subacute onset of neurological symptoms, which can include lethargy, confusion, and delirium. However, the commonest symptoms are fever (90%), headache (81%), psychiatric symptoms (71%), convulsions (67%), vomiting (46%), weakness (33%), and memory loss (24%) [6]. HSVE has no diagnostic features. Focal neurological deficits, lymphocytic pleocytosis in the CSF, and neuroimaging-detected changes may initially be lacking. During evaluation, it is important to exclude other possible causes of encephalitis [7]. HSV-1 encephalitis has high morbidity and mortality. The mortality rate in untreated patients is approximately 70%, and normal neurological function is not restored to 97% of the surviving patients, who experience sequelae [8–11]. The complications of HSVE can be divided into the acute effects, including cerebral edema, intracranial hypertension, cerebral herniation and seizures, and chronic complications, which depend upon the areas affected and can also include anti-NMDA (N-methyl-d-aspartate) receptor encephalitis.

In most patients, encephalitis develops monophasically. In some cases, the patients return to their doctors after treatment, with an apparent clinical relapse. These patients can be of any age, with a large variety of neurological manifestations, including behavioral or personality changes, memory loss, and seizures. Negative prognostic factors are advanced age, coma or a depressed level of consciousness at onset, and a delayed specific antiviral treatment. Normal electroencephalography predicts survival independently of possible confounders [12]. An early diagnosis and timely treatment with acyclovir are very important in optimizing the outcome [13]. Delayed treatment with acyclovir is the negative prognostic factor that can be changed most easily. The prognosis can also be affected by immunosuppression, severe comorbidities, a history of alcohol abuse, lack of fever, and CSF leucocytes <10/ml. The guidelines strongly recommend that acyclovir therapy be administered as soon as possible when HSVE is suspected [14]. Renal function, as in our patient, should always be assessed and the acyclovir dose adjusted according to the following scheme. A delayed or wrong diagnosis or delayed or inappropriate therapy may also entail medical liability [15]. In these cases, it is very important that the pathologists recognize HSVE to pursue as correctly as possible every case of suspicious medical liability and to make a final assessment of the sequelae of this disease.
